# Supplementary material for: GenACO a multi-objective cached data offloading optimization based on genetic algorithm and ant colony optimization
Source: PeerJ Comput Sci. 2021 Sep 28;7:e729. doi: 10.7717/peerj-cs.729 (PMC8507473; doi:10.7717/peerj-cs.729)
Supplement: Supplemental Information 1 [file peerj-cs-07-729-s001.docx]

Appendix Table 1. Cached data offloading performance comparison by single ACO.

| **Single ACO** | | | | | | | | |
| --- | --- | --- | --- | --- | --- | --- | --- | --- |
| **ith.** | Scenario-1a | | Scenario-1b | | Scenario-1c | | Scenario-1d | |
|  | Fx | ch.dt | Fx | ch.dt | Fx | ch.dt | Fx | ch.dt |
| 1 | 0.4761 | 13 | 0.465 | 12 | 0.4843 | 12 | 0.435 | 15 |
| 2 | 0.4928 | 10 | 0.4374 | 15 | 0.4309 | 13 | 0.4405 | 15 |
| 3 | 0.5157 | 12 | 0.4374 | 15 | 0.4521 | 13 | 0.435 | 15 |
| 4 | 0.4802 | 13 | 0.4614 | 13 | 0.4409 | 14 | 0.435 | 15 |
| 5 | 0.4875 | 13 | 0.4405 | 15 | 0.4382 | 15 | 0.435 | 15 |
| 6 | 0.499 | 12 | 0.4305 | 14 | 0.447 | 13 | 0.4405 | 15 |
| 7 | 0.4959 | 11 | 0.4305 | 14 | 0.4382 | 15 | 0.435 | 15 |
| 8 | 0.4715 | 13 | 0.4374 | 15 | 0.4386 | 13 | 0.435 | 15 |
| 9 | 0.4926 | 12 | 0.4609 | 13 | 0.4382 | 15 | 0.435 | 15 |
| 10 | 0.5085 | 12 | 0.4305 | 14 | 0.449 | 13 | 0.435 | 15 |
| 11 | 0.4848 | 13 | 0.4667 | 14 | 0.4409 | 13 | 0.435 | 15 |
| 12 | 0.4811 | 13 | 0.4604 | 14 | 0.447 | 14 | 0.435 | 15 |
| 13 | 0.4533 | 14 | 0.4374 | 14 | 0.4382 | 15 | 0.435 | 15 |
| 14 | 0.4765 | 12 | 0.4563 | 14 | 0.447 | 14 | 0.4305 | 14 |
| 15 | 0.5293 | 11 | 0.4417 | 14 | 0.4378 | 14 | 0.4405 | 15 |
| 16 | 0.4952 | 11 | 0.4708 | 12 | 0.4309 | 13 | 0.435 | 15 |
| 17 | 0.5377 | 11 | 0.4374 | 15 | 0.4309 | 12 | 0.435 | 15 |
| 18 | 0.4896 | 12 | 0.4405 | 15 | 0.4409 | 14 | 0.435 | 15 |
| 19 | 0.4759 | 14 | 0.4305 | 14 | 0.4409 | 14 | 0.435 | 15 |
| 20 | 0.5018 | 12 | 0.4531 | 14 | 0.4512 | 13 | 0.4405 | 15 |
| 21 | 0.4785 | 13 | 0.4382 | 15 | 0.4382 | 15 | 0.435 | 15 |
| **AVG** | 0.4916 | 12.24 | 0.4459 | 14.05 | 0.4429 | 13.67 | 0.4358 | 14.95 |
| **BEST** | 0.4533 | 14 | 0.4374 | 15 | 0.4382 | 15 | 0.435 | 15 |
| **WORST** | 0.4928 | 10 | 0.4708 | 12 | 0.4843 | 12 | 0.4305 | 14 |

Appendix Table 2. Cached data offloading performance comparison by single GA.

| **Single GA** | | | | | | | | |
| --- | --- | --- | --- | --- | --- | --- | --- | --- |
| **ith.** | Scenario-2a | | Scenario-2b | | Scenario-2c | | Scenario-2d | |
|  | Fx | ch.dt | Fx | ch.dt | Fx | ch.dt | Fx | ch.dt |
| 1 | 0.4653 | 11 | 0.4105 | 10 | 0.4928 | 10 | 0.4063 | 13 |
| 2 | 0.4653 | 11 | 0.4105 | 10 | 0.4292 | 12 | 0.4063 | 13 |
| 3 | 0.4653 | 11 | 0.3921 | 11 | 0.4292 | 12 | 0.4063 | 13 |
| 4 | 0.4653 | 11 | 0.4269 | 12 | 0.4292 | 12 | 0.4063 | 13 |
| 5 | 0.4653 | 11 | 0.4269 | 12 | 0.3851 | 12 | 0.4063 | 13 |
| 6 | 0.4653 | 11 | 0.4126 | 13 | 0.3851 | 12 | 0.4063 | 13 |
| 7 | 0.4653 | 11 | 0.4126 | 13 | 0.3851 | 12 | 0.4063 | 13 |
| 8 | 0.4653 | 11 | 0.4126 | 13 | 0.3851 | 12 | 0.4063 | 13 |
| 9 | 0.4653 | 11 | 0.4126 | 13 | 0.3851 | 12 | 0.4063 | 13 |
| 10 | 0.4653 | 11 | 0.4126 | 13 | 0.3851 | 12 | 0.4063 | 13 |
| 11 | 0.4653 | 11 | 0.4126 | 13 | 0.3851 | 12 | 0.4063 | 13 |
| 12 | 0.4653 | 11 | 0.4126 | 13 | 0.3851 | 12 | 0.4063 | 13 |
| 13 | 0.4653 | 11 | 0.3855 | 14 | 0.3776 | 13 | 0.4063 | 13 |
| 14 | 0.4653 | 11 | 0.3855 | 14 | 0.3776 | 13 | 0.4063 | 13 |
| 15 | 0.4653 | 11 | 0.3855 | 14 | 0.3776 | 13 | 0.4063 | 13 |
| 16 | 0.4653 | 11 | 0.3855 | 14 | 0.3776 | 13 | 0.4063 | 13 |
| 17 | 0.4653 | 11 | 0.3855 | 14 | 0.3776 | 13 | 0.4063 | 13 |
| 18 | 0.4653 | 11 | 0.3855 | 14 | 0.3776 | 13 | 0.4063 | 13 |
| 19 | 0.4653 | 11 | 0.3855 | 14 | 0.3776 | 13 | 0.3999 | 13 |
| 20 | 0.4653 | 11 | 0.3855 | 14 | 0.3776 | 13 | 0.3999 | 13 |
| 21 | 0.4653 | 11 | 0.3855 | 14 | 0.3776 | 13 | 0.3999 | 13 |
| **AVG** | 0.4653 | 11 | 0.4012 | 12.95 | 0.3933 | 12.33 | 0.4054 | 13 |
| **BEST** | 0.4653 | 11 | 0.3855 | 14 | 0.3776 | 13 | 0.3999 | 13 |
| **WORST** | 0.4653 | 11 | 0.4105 | 10 | 0.4928 | 10 | 0.4063 | 13 |

Appendix Table 3. Cached data offloading performance by CGACA.

| **Framework : CGACA** | | | | | | |
| --- | --- | --- | --- | --- | --- | --- |
| **ith.** | **stagnant:** ith=5 | | **normal** | | **stagnant:** ith=13 | |
|  | Fx | ch.dt | Fx | ch.dt | Fx | ch.dt |
| 1 | 0.4995 | 12 | 0.5262 | 12 | 0.4852 | 13 |
| 2 | 0.4995 | 12 | 0.5262 | 12 | 0.4852 | 13 |
| 3 | 0.4995 | 12 | 0.5262 | 12 | 0.4844 | 12 |
| 4 | 0.4995 | 12 | 0.5164 | 10 | 0.4852 | 13 |
| 5 | 0.4995 | 12 | 0.5169 | 7 | 0.4852 | 13 |
| 6 | 0.4995 | 12 | 0.5262 | 12 | 0.4852 | 13 |
| 7 | 0.4732 | 12 | 0.4962 | 8 | 0.4852 | 13 |
| 8 | 0.5084 | 11 | 0.5262 | 12 | 0.4749 | 11 |
| 9 | 0.4852 | 12 | 0.5161 | 11 | 0.4852 | 13 |
| 10 | 0.4627 | 14 | 0.5051 | 10 | 0.4852 | 13 |
| 11 | 0.5023 | 12 | 0.5107 | 12 | 0.4852 | 13 |
| 12 | 0.5257 | 12 | 0.5018 | 12 | 0.4852 | 13 |
| 13 | 0.4816 | 12 | 0.499 | 12 | 0.4852 | 13 |
| 14 | 0.4704 | 13 | 0.4975 | 11 | 0.5138 | 12 |
| 15 | 0.4672 | 14 | 0.4907 | 9 | 0.5085 | 12 |
| 16 | 0.4627 | 13 | 0.499 | 12 | 0.4772 | 14 |
| 17 | 0.5208 | 11 | 0.499 | 12 | 0.4873 | 12 |
| 18 | 0.4661 | 13 | 0.499 | 12 | 0.4999 | 13 |
| 19 | 0.4978 | 13 | 0.48 | 11 | 0.4476 | 14 |
| 20 | 0.4869 | 12 | 0.499 | 12 | 0.4705 | 12 |
| **AVG** | 0.4904 | 12.3 | 0.5079 | 11.05 | 0.4851 | 12.75 |
| **BEST** | 0.4672 | 14 | 0.499 | 12 | 0.4476 | 14 |
| **WORST** | 0.5208 | 11 | 0.5169 | 7 | 0.4749 | 11 |
| **TIME** | 40.21 sec | | 31.29 sec | | 34.77 sec | |

Appendix Table 4. A comparison of cached data offloading performance achieved by CGACA and GenACO.

| **ith.** | **CGACA** | | **cyclic GenACO** | | **non-cyclic GenACO** | |
| --- | --- | --- | --- | --- | --- | --- |
|  | Fx | ch.dt | Fx | ch.dt | Fx | ch.dt |
| 1 | 0.4779 | 12 | 0.4466 | 14 | 0.4305 | 14 |
| 2 | 0.4779 | 12 | 0.4466 | 14 | 0.4305 | 14 |
| 3 | 0.4779 | 12 | 0.4458 | 12 | 0.435 | 15 |
| 4 | 0.4779 | 12 | 0.4466 | 14 | 0.435 | 15 |
| 5 | 0.4779 | 12 | 0.4466 | 14 | 0.435 | 15 |
| 6 | 0.4779 | 12 | 0.4305 | 14 | 0.4309 | 12 |
| 7 | 0.4958 | 11 | 0.4305 | 14 | 0.435 | 15 |
| 8 | 0.5175 | 11 | 0.435 | 15 | 0.435 | 15 |
| 9 | 0.498 | 12 | 0.435 | 15 | 0.4305 | 14 |
| 10 | 0.4845 | 13 | 0.435 | 15 | 0.435 | 15 |
| 11 | 0.5055 | 11 | 0.435 | 15 | 0.435 | 15 |
| 12 | 0.4722 | 13 | 0.435 | 15 | 0.435 | 15 |
| 13 | 0.523 | 12 | 0.4466 | 14 | 0.435 | 15 |
| 14 | 0.4882 | 13 | 0.4382 | 14 | 0.435 | 15 |
| 15 | 0.477 | 13 | 0.4382 | 14 | 0.435 | 15 |
| 16 | 0.4818 | 13 | 0.4466 | 14 | 0.435 | 15 |
| 17 | 0.4698 | 13 | 0.4382 | 14 | 0.435 | 15 |
| 18 | 0.4374 | 15 | 0.4466 | 14 | 0.435 | 15 |
| 19 | 0.501 | 12 | 0.4382 | 14 | 0.435 | 15 |
| 20 | 0.48 | 12 | 0.4382 | 14 | 0.435 | 15 |
| 21 | 0.4817 | 13 | 0.4305 | 14 | 0.435 | 15 |
| **AVG** | 0.4848 | 12.33 | 0.4395 | 14.14 | 0.4342 | 14.71 |
| **BEST** | 0.4374 | 15 | 0.435 | 15 | 0.435 | 15 |
| **WORST** | 0.5175 | 11 | 0.4458 | 12 | 0.4305 | 14 |
| **TIME** | 48.44 sec | | 25.34 sec | | 29.84 sec | |
